# Comparative ribosome profiling uncovers a dominant role for translational control in *Toxoplasma gondii*

**DOI:** 10.1186/s12864-017-4362-6

**Published:** 2017-12-11

**Authors:** Musa A. Hassan, Juan J. Vasquez, Chew Guo-Liang, Markus Meissner, T. Nicolai Siegel

**Affiliations:** 1 Division of Infection and Immunity, The Roslin Institute, University of Edinburgh, Edinburgh, UK; 2 The Centre for Tropical Livestock Genetics and Health, The Roslin Institute, University of Edinburgh, Edinburgh, UK; 3 Research Centre for Infectious Diseases, University of Wuerzburg, Wuerzburg, 97080 Germany; 4 Computational Biology Program, Basic Sciences and Public Health Sciences Division, Fred Hutchinson Cancer Research Centre, Seattle, WA 98105 USA; 5 Wellcome Centre for Molecular Parasitology, University of Glasgow, Glasgow, UK; 6 Department of Veterinary Sciences, Experimental Parasitology, Ludwig-Maximilians-Universität, München, 80802 Munich, Germany; 7 Biomedical Center Munich, Physiological Chemistry, Ludwig-Maximilians-Universität München, Planegg-Martinsried, 82152 Germany; 8 Present address: Department of Biological Engineering, Massachusetts Institute of Technology, Cambridge, MA 02139 USA

**Keywords:** Ribosome profiling, RNA-sequencing, Translation efficiency, *Toxoplasma gondii*, Apicomplexan

## Abstract

**Background:**

The lytic cycle of the protozoan parasite *Toxoplasma gondii,* which involves a brief sojourn in the extracellular space, is characterized by defined transcriptional profiles. For an obligate intracellular parasite that is shielded from the cytosolic host immune factors by a parasitophorous vacuole, the brief entry into the extracellular space is likely to exert enormous stress. Due to its role in cellular stress response, we hypothesize that translational control plays an important role in regulating gene expression in *Toxoplasma* during the lytic cycle. Unlike transcriptional profiles, insights into genome-wide translational profiles of *Toxoplasma gondii* are lacking.

**Methods:**

We have performed genome-wide ribosome profiling, coupled with high throughput RNA sequencing, in intracellular and extracellular *Toxoplasma gondii* parasites to investigate translational control during the lytic cycle.

**Results:**

Although differences in transcript abundance were mostly mirrored at the translational level, we observed significant differences in the abundance of ribosome footprints between the two parasite stages. Furthermore, our data suggest that mRNA translation in the parasite is potentially regulated by mRNA secondary structure and upstream open reading frames.

**Conclusion:**

We show that most of the *Toxoplasma* genes that are dysregulated during the lytic cycle are translationally regulated.

**Electronic supplementary material:**

The online version of this article (10.1186/s12864-017-4362-6) contains supplementary material, which is available to authorized users.

## Background

All living organisms are constantly exposed to a variety of biological stress, which may include limiting nutrient availability, oxidative stress, temperature shock, DNA damage and infection. Consequently, organisms show remarkable regulatory plasticity that allows them to thrive under different, sometimes harsh, environmental conditions [1, 2]. Historically, due to the relative ease of obtaining global transcript abundance estimates, most studies quantify fluctuations in mRNA abundance to gain insights into organismal response to stress [3, 4]. However, the catalogue of expressed genes and proteins is modulated at various steps, including mRNA splicing, export, stability, translation, and protein degradation [5]. Consequently, transcript abundance rarely mirrors cellular protein levels [6]. Although the relative contribution of each of these steps in the gene-expression pathway is equivocal, mRNA translation is known to play a significant role in modulating cellular protein levels [7, 8]. Indeed, translational control of gene expression is known to provide opportunities for controlling spatial and temporal protein distribution [9]. Furthermore, because most eukaryotic mRNAs can be detected in cells at least 2 h after expression [10], compared to de novo transcription, regulating the translation of the available mRNAs provides a mechanism to rapidly adjust cellular protein levels in response to drastic changes in the environment or developmental stages [11, 12]. In fact, most translationally regulated mRNAs are known to encode proteins that regulate important cellular processes such as stress response, development, and synaptic transmission [7].

*Toxoplasma gondii* is an obligate intracellular apicomplexan that infects virtually all warm-blooded vertebrates. In the definitive feline host, *Toxoplasma* undergoes sexual recombination, but reverts to asexual reproduction in the intermediate host, which includes humans. Asexual reproduction in *Toxoplasma* is characterized by the rapidly dividing tachyzoite stage. However, in response to host-derived stress factors, such as immune response, the rapidly dividing tachyzoites convert to the semi-dormant encysted bradyzoites and establish lifelong chronic infections in the central nervous system and muscle tissues of the host [13, 14]. Establishment of chronic infection is important for the re-entry of the parasite into the definitive host, and for horizontal transmission within intermediate hosts, through the predation and consumption of food products from chronically infected hosts, respectively [15]. The tachyzoite-to-bradyzoite conversion reportedly mirrors a stress response [2] and does not only involve significant changes in the parasite physiology and morphology, but also is accompanied by altered gene expression profiles [16]. During the lytic cycle the parasite invades a host cell, replicates, and then lyses out of the host cell before infecting a new host cell. This process temporarily exposes the parasite to the extracellular milieu. The extracellular viability of the parasite is reported to decrease dramatically between 6 and 12 h after egress [17], indicating the level of biological stress induced on the parasite by host factors. Indeed, transcriptional data on most *Toxoplasma* strains have revealed stage-specific expression of several genes, such as surface antigens, stress response genes, virulence genes, and metabolic enzymes [13, 18, 19]. Consequently, regulating transcript abundance, and by extension their protein products, is key in regulating *Toxoplasma* developmental stages and intercellular transmission.

Translational regulation of gene expression has emerged as a key factor in the biology of apicomplexan parasites [20–23]. In *Plasmodium,* translational regulation is reported to modulate stage conversion and host-parasite interactions [20, 21]. For example, while *Pb2* transcripts, a surface antigen, can be detected in *Plasmodium berghei* female gametocytes, the translation of *Pb2* mRNA occurs only when the parasite is in the mosquito gut [24]. In *Toxoplasma*, genetic perturbation of the eukaryotic elongation factor 2 alpha (eIF2α), an important component of the translation initiation complex, affects extracellular viability. Phosphorylated eIF2α is essential for transferring the initial methionyl tRNA (Met-tRNAi) to the 40S pre-initiation complex [25]. However, when phosphorylated at a regulatory serine (serine-51), eIF2α is unable to activate Met-tRNAi and global translation is diminished [25]. *Toxoplasma* parasites expressing eIF2 (TgIF2α) with a mutation on the regulatory serine (serine-71) are reported to exhibit decreased extracellular viability [26]. Lower expression levels of *eIF4,* another translation initiation factor, has been observed in bradyzoites and attenuated *Toxoplasma* strains [2, 23, 24, 26]. Finally, the endoplasmic reticulum (ER) stress response in *Toxoplasma* is characterized by preferential translation of a subset of genes, including the transcriptional regulator *AP2* [23, 27]. This is particularly important since the integrity of the parasite ER is pivotal for the proper folding of essential proteins required for parasite invasion, immune evasion and the establishment of chronic infection [24]. Thus, it is plausible that the viability, pathogenesis, and transmission of *Toxoplasma* are dependent on its ability to recognize and translationally respond to host-derived stress.

Genome-wide insights on translational control and the underlying molecular factors that regulate mRNA translation in *Toxoplasma* are largely unknown. Here, we access the translational landscape in *Toxoplasma gondii* and determine its impact on intercellular parasite transmission. To do this, we have used ribosome profiling to capture genome-wide translational profiles of intracellular and extracellular *Toxoplasma* parasites infecting human foreskin fibroblasts. Our data reveal a putative role for translational control in regulating parasite gene expression during the lytic cycle. Additionally, our data revealed variable translational efficiency of several dysregulated *Toxoplasma* mRNAs, such as the mRNAs encoding dense granules, which are known to be spatially secreted during the lytic cycle. Finally, our data suggest that that mRNA secondary structure, putatively affect mRNA translation in *Toxoplasma*. These results not only provide greater insights into *Toxoplasma* gene regulation, but also provide a resource and template for elucidating the function of translational control in *Toxoplasma* biology. Finally, the ribosome footprints, will provide an additional resource for annotating *Toxoplasma* transcript features.

## Results

### Generation of mRNA profiles and ribosome footprints in intracellular and extracellular *Toxoplasma*

**Fig. 1:**
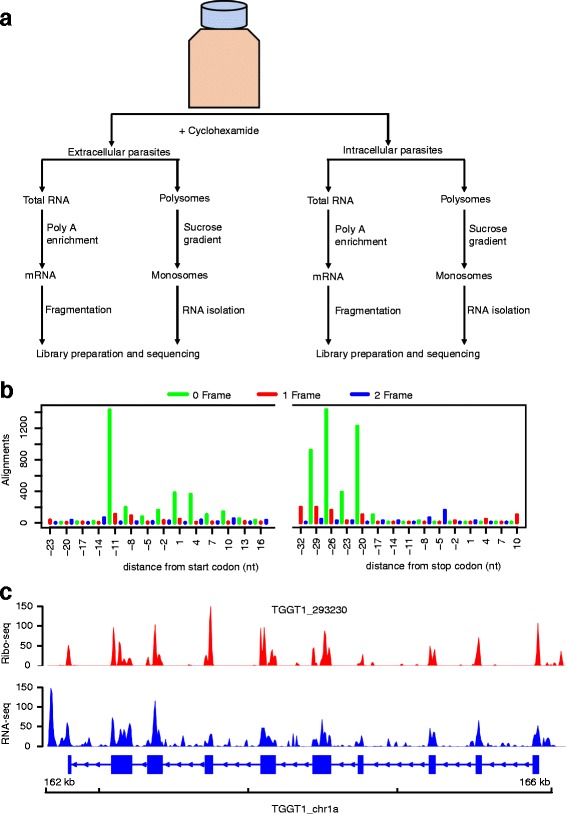
Ribosome profiling of *Toxoplasma gondii*. **a** The experimental design. Cyclohexamide was added to the flasks for ~10 min prior to collecting the medium containing extracellular parasites and the host-cell monolayer was syringe lysed to release intracellular parasites. Chemically fragmented mRNA and sucrose gradient fractionated monosomes were used to prepare RNA-seq and Ribo-seq libraries, respectively, in parallel. **b** P-site tracks, colour-coded per frame, were created for all annotated exons in RiboTaper. **c** Ribo-seq (red peaks) and RNA-seq (blue peaks) read pile-up, presented as Fragment per kilobase exon per million reads (FPKM), on a multi-exonic gene. Exons are shown as blue blocks while introns are represented by blue lines

To investigate genome-wide transcriptional and translational status in *Toxoplasma* during the lytic cycle, we performed RNA sequencing (RNA-seq) and ribosome profiling on two biological replicates of extracellular and intracellular parasites as previously described [4] (the experimental layout is depicted in Fig. 1a).

The basic concept of ribosome profiling is that actively translated mRNAs are protected from ribonucleases by the decoding ribosomes. However, other classes of RNA-binding proteins can protect mRNA from nucleases. Therefore, the presence of sequencing reads derived from nuclease-resistant RNA fragments does not necessarily infer active translation. Since ribosomes decode mRNA by reading 3-nucleotides (3-nt) at a time, 3-nt periodicity on ribosome footprints is often used to distinguish ribosome protected RNA from other classes of nuclease resistant RNAs [3, 28–30]. Therefore, to increase coverage, we pooled ribosome-protected RNA footprints from the two biological replicates for each sample, and used sub-codon resolution to call high confidence translated open reading frames (ORFs) in canonical *Toxoplasma* coding sequences. To do this, we used RiboTaper, a ribosome profiling analysis program that defines the peptidyl-site (P-site; the second tRNA entry site linked to the growing polypeptide chain) of ribosome-protected RNA sequencing (Ribo-seq) reads mapping over annotated transcripts [3]. Henceforth, unless otherwise stated, all analyses on extracellular or intracellular samples are based on pooled Ribo-seq or RNA-seq data. Because 3-nt periodicity often vary between different Ribo-seq read lengths, we performed sub-codon resolution on 25–30-nt reads, which is within the range of 80S ribosome-protected RNA lengths [5]. We observed a strong 3-nt periodicity in 29-nt footprints, with up to 12-nt upstream of the AUG start site covered by ribosome footprints (12-nt offset) (Fig. 1b). Similar offsets were obtained using Riboprofiling [31], a Bioconductor package for processing Ribo-seq data (Additional file 1: Figure S1). Unlike RNA-seq reads that sometimes contain reads aligning to intronic regions, ribosome footprints mapped predominantly to annotated *Toxoplasma* protein coding regions (Fig. 1c). Therefore, the ribosome footprints in the current experiment are mostly derived from ribosome-protected nuclease-resistant mRNA fragments and can be used to accurately quantify translation in *Toxoplasma*.

### Ribosome profiling confirms translation of annotated CDSes and identifies novel translated ORFs in *Toxoplasma*

**Fig. 2:**
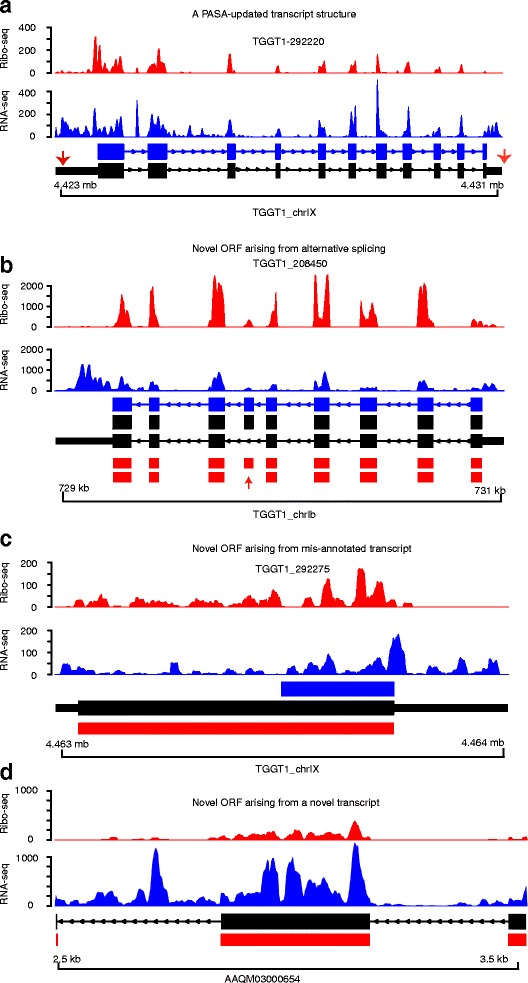
Ribosome profiling reveals novel and annotated open reading frames. **a** Shown are examples of PASA-updated *Toxoplasma* transcript structure annotations (Black) and the corresponding current ToxoDB transcript annotation (Blue). A) The PASA-updated transcript is due to the addition of 5′/3′UTRs (red arrows). Prediction of ORFs based on the PASA-updated transcript structures in RiboTaper identified canonical and novel ORFs. The novel ORFs were mainly due to; **b** alternative splicing of annotated transcripts (skipped exon, red arrow), **c** potentially mis-annotated transcripts structures, and **d** novel transcripts. The RiboTaper-predicted ORFs in B-D are presented as red blocks. In all the figures, Ribo-seq and RNA-seq read coverage on each transcript is shown in Fragment per kilobase exon per million reads (FPKM)

RNA-seq alone cannot distinguish translated from non-translated transcripts. Additionally, it is not clear whether some annotated non-coding RNAs contain translated small open reading frames. These problems are exacerbated in non-model organisms, such as *Toxoplasma*, with incompletely annotated genomes. Because Ribo-seq captures ribosome-engaged mRNAs, it is often used to not only estimate the translation efficiencies of annotated coding regions, but also to identify novel translated ORFs. Consequently, we used RiboTaper, as previously described [3], to identify translated ORFs based on 3-nt periodicity and P-site positions in the expressed *Toxoplasma* genes. Because the current annotation of *Toxoplasma* gene structures (ToxoDB.org; GT1 v28 [32]) is incomplete, and RiboTaper classifies ORFs based on known coding regions, we initially used RNA-seq reads (~500 million paired-end reads from this and a parallel study [33]) to update GT1 gene structures. To do this, we performed genome-guided transcript assembly using Trinity [34], followed by transcript structure resolution using the Program to Assemble Spliced Alignments (PASA) [35], as previously described [36]. Subsequently, we updated the structures of 6442 transcripts, mostly due to the addition of 5′ and 3′ UTRs (mean lengths of 435-nt and 508-nt, respectively) (Fig. 2a). Next, we used RiboTaper and identified 4224 ORFs in 4195 genes based on the updated transcript structures. Noteworthy, the identification of ORFs in RiboTaper is based on codon resolution on the Ribo-seq reads that map to annotated transcript features rather than the simple presence of ribosome footprints. Thus, the number of translated ORFs identified by this approach may be lower than the actual number of genes with ribosome footprints. Besides canonical ORFs, we identified 172 novel ORFs, mainly due to the alternative splicing of annotated transcripts (Fig. 2b), PASA-updated new transcripts structures (Fig. 2c), or novel transcripts (Fig. 2d). Therefore, by using ribosome footprints, we not only provide a greater resolution of the canonical ORFs to include alternative isoforms, but also identify novel ORFs in *Toxoplasma* (GT1)*.*

### Steady-state mRNA and translation efficiency in intracellular and extracellular parasites

We sought to evaluate global translational divergence in intracellular versus extracellular type I *Toxoplasma* parasites. Initially, we used HTSeq [37] to obtain raw read counts from the uniquely mapped RNA-seq and Ribo-seq reads, which were then normalized (Normalized Read Counts, NRC) in DESeq2 [38] to adjust for variation in sequencing depths across samples. Even though some genes were lowly expressed, approximately 7065 genes (83% of the ~8460 genes annotated in the GT1 genome (v28) were expressed (average RNA-seq NRC > 5 across samples) (Additional file 2 A). Of the expressed genes, 6508 had ribosome footprints (average Ribo-seq NRC > 5 across samples), suggesting that 557 transcripts are non-coding or poorly translated in our parasite populations (Additional file 2 B). Interestingly, 274/557 (> 50% of the potentially non-coding or poorly translated genes) had an average RNA-seq NRC > 10 (mean NRC = 41.18; SEM = ±3.48), suggesting that these genes are expressed above background levels (which we arbitrarily set at NRC < 5) but are either translationally repressed in these parasite populations or non-coding. The protein products for most of these 274 genes are annotated in ToxoDB as “hypothetical”, but also included the KRUF proteins, which are encoded from a highly expanded gene family in the GT1 strain [39]. Also included in the 274 poorly translated or non-coding genes was the *Toxoplasma* translation initiation factor 2 (TgIF2K-C), which is required for the parasites’ response to intracellular glutamine starvation in human cells [40]. These 274 transcripts were functionally enriched for, among others, “cell adhesion” (Bonferroni *P* value = *3.58e-4*) and “microtubule motor activity” (Bonferroni *P* value = *9.96e-4*). Although they are included in the current GT1 genome annotation, 27 of the 274 genes did not have any corresponding proteomic data in ToxoDB [32], suggesting that they are non-coding. On the other hand, 83 transcripts exhibited low abundance with an average RNA-seq NRC < 5 (mean NRC = 3.14; SEM = ±0.14), but had average Ribo-seq NRC > 5 (mean NRC = 13.17; SEM = ±3.52) (Additional file 2 C). These 87 genes included the SAG-related sequence (SRS) gene family that are implicated in *Toxoplasma* virulence in mice [41].

**Fig. 3:**
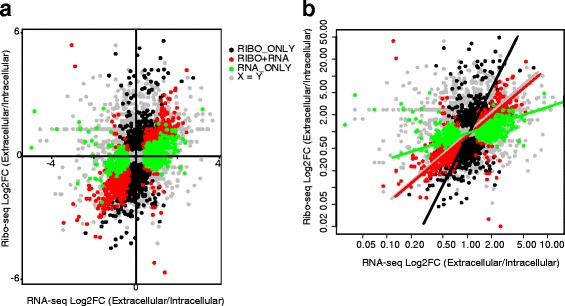
Translation regulation is predominant in *Toxoplasma gondii*. **a** Although most transcripts showed no differential regulation (grey dots *n = 4332*) between the intracellular and extracellular parasites, several dysregulated genes were significantly (Benjamini–Hochberg *FDR ≤ 0.1*) regulated at the level of mRNA abundance only (green dots, RNA-ONLY, *n = 650*), ribosome occupancy only (black dots, RIBO-ONLY; *n = 1339*), and at the level of both mRNA abundance and ribosome occupancy (red dots, RIBO + RNA, *n = 899*). Differences in translational efficiency in intracellular versus extracellular parasites were calculated in Ribodiff on transcripts with average RNA-seq DESeq2 normalized read counts ≥ 5. **b** The slope for red spots approached 1 (x = y), indicating equal fold changes in gene regulation at the level of mRNA abundance and ribosome occupancy between the intracellular and extracellular parasites (standardized major-axis estimation; SMA). Significant (*P < 2.22e-16*) divergence of black and red slopes demonstrate differences in gene regulation at the level of ribosome occupancy (RIBO-ONLY) and mRNA abundance (RNA-ONLY), respectively

Next, we compared differences in mRNA abundance and ribosome occupancy between the intracellular and extracellular parasites. Using a Benjamini-Hochberg False Discovery Rate (FDR) ≤ 10%, we identified three classes of differentially regulated genes: 1) 891 genes that varied both at the level of transcript abundance and ribosome occupancy i.e. concordant (RNA + RIBO) (Additional file 2 D), 2) 645 genes that varied only at the levels of mRNA abundance (RNA-ONLY) (Additional file 2 E), and 3) 1324 genes that varied only at the level of ribosome occupancy (RIBO-ONLY) (Fig. 3a and Additional file 2 F), indicating that many of the genes that are dysregulated in *Toxoplasma* during the lytic cycle are regulated at the translational level. To determine the overall contribution of translation in regulating gene expression during *Toxoplasma’s* lytic cycle, we used a standardized major-axis estimation (SME) [42] analysis to calculate the slopes of fold changes in RNA-seq or Ribo-seq NRCs between intracellular and extracellular parasites. Unlike the RIBO + RNA transcripts, where the slope approached 1 (slope = 1.15), indicating the co-occurrence of changes in transcript abundance and ribosome occupancy, the slope for RIBO-ONLY transcripts (slope = 2.87) was significantly (*P* value *< 2.22e-16*) greater than 1 (Fig. 3b), confirming that many differences in gene expression between the intracellular and extracellular parasites occur at the translation level.

Next, we calculated differences in translation efficiency (TE) for each expressed transcript between extracellular and intracellular parasites using Ribodiff [43]. At a Benjamini-Hochberg FDR ≤ 10%, we identified differential TE in 834 genes in intracellular versus extracellular parasites (Additional file 2 G). Because of the potential variation in mRNA between intracellular and extracellular parasites, which in the absence of a spike-in control during RNA sequencing may skew the data, we complemented the ribodiff protocol by ranking the genes based on the z-scores of TE in each parasite population. We considered genes with RNA-seq NRC ≥ 5 (7065 genes) and at least two standard deviations above or below the mean TE in each population as translationally up- or down- regulated, respectively. By this metric, 868 genes were translationally down-regulated while 119 genes were up-regulated in intracellular parasites. On the other hand, 1004 and 236 genes were down- and up-regulated, respectively, in extracellular parasites. Of the dysregulated genes, 344 and 556 genes were exclusively dysregulated in intracellular and extracellular parasites, respectively (not deviating from the mean or dysregulated in the opposite directions in the two populations, e.g. up-regulated in intracellular but down-regulated in extracellular parasites). The “sporozoite development protein (TGGT1_257010)” and “BT1 family protein (TGGT1_236020)” genes were the most down- (z-score = −5.0; Log_2_TE = −6.03) and up-regulated (z-score = 4.79; Log_2_TE = 4.15), respectively, in intracellular parasites. In extracellular parasites, “the transporter, major facilitator family protein (TGGT1_266870)” and “CMGC kinase, CDK family (TGGT1_253580)” genes were the most down- (z-score = −5.85; Log_2_TE = 6.43) and up-regulated (z-score = 6.13; Log_2_TE = 5.55), respectively. Although dense granules are secreted by intracellular parasites [44], the translation efficiency for genes encoding these proteins (GRA1, GRA4, and GRA7) was up-regulated in extracellular parasites. Additionally, the translation of genes encoding the alveolin domain-containing inner membrane complex (IMC) proteins (IMC1, IMC4, IMC6, and IMC10), which are required during intracellular *Toxoplasma* cell division [45], were up-regulated in extracellular parasites.

### Most *Toxoplasma* transcripts contain open reading frames (ORFs) at their 5′ untranslated regions

**Fig. 4:**
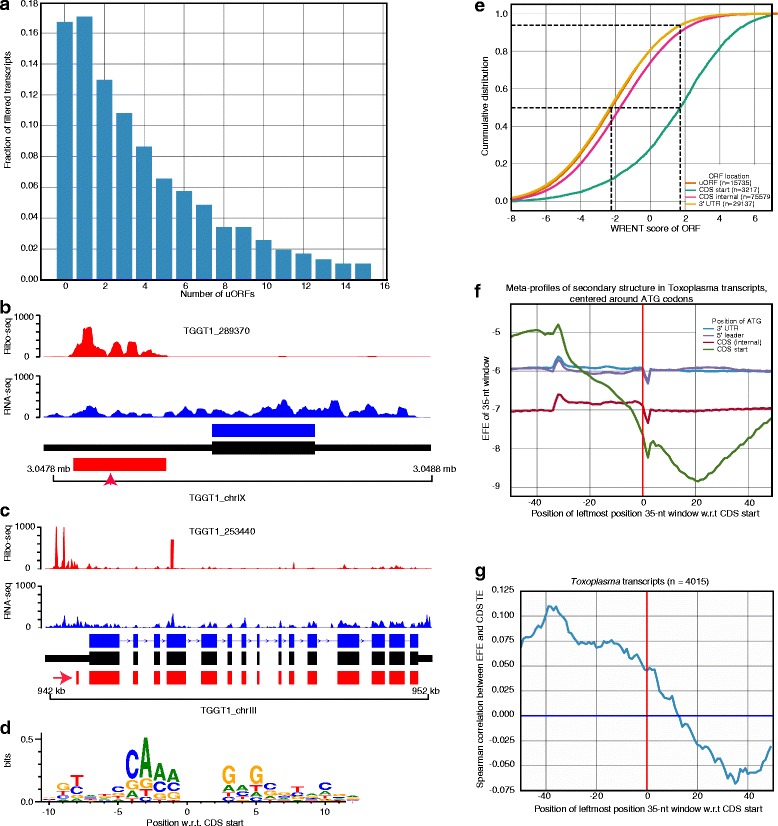
Open reading frames in 5′ leader sequences (uORFs) are abundant in *Toxoplasma*. **a** Based on the presence of a start and a downstream in-frame stop codon, some *Toxoplasma* transcripts contain more than 14 non-overlapping uORFs. **b**-**c** Individual *Toxoplasma* transcripts with an upstream translated uORFs (red arrow) exhibit reduced ribosome occupancy at the cognate canonical protein coding sequences. Ribo-seq (red peaks) and RNA-seq (blue peaks) read pile-up on ToxoDB (blue) and PASA-updated (black) transcript structures. **d** Weighted position-specific scoring matrices (PSSMs) around (±10 nucleotides) annotated ATG of transcripts lacking uORFs. Vertical axis represents weighted relative entropy (WRENT). **e** Cumulative WRENT scores around ATG start codons along the coding transcripts. Dotted lines indicate median WRENT scores of uORF and CDS, and the proportion of uORFs (~90%) with WRENT scores less than the median CDS WRENT score. **f** Predicted secondary structure ensemble free energies (EFEs) in a sliding 35-nt window on 5′ UTRs, CDSes, and 3′ UTRs. A more negative EFE indicates more stable secondary structures. **g** The secondary structure EFE correlates with translation efficiency in CDSes. See also Additional file 3: Figure S2 and Additional file 4: Table S1

Besides translation at canonical protein coding sequences (CDSes), ribosome profiling can reveal novel coding sequences, including coding sequences at the 5′ and 3′ untranslated regions (upstream and downstream ORFs, uORFs and dORFs, respectively) [3, 28]. Because the prevalence and translation regulatory potential of uORFs is largely unknown in *Toxoplasma*, we used a support vector classifier [29] to identify translated uORFs. Based on the presence of a start and an in-frame downstream stop codon, we observed a high prevalence of uORFs in *Toxoplasma*, with some transcripts having > 4 non-overlapping uORFs (Fig. 4a). From 4577 transcripts with annotated 5′ UTRs of lengths ≥ 20-nt, we identified uORFs in 3348 (73%). Similar abundance of uORFs has also been reported in *Plasmodium falciparum* [21]. We filtered the transcripts further to 2770 (translated uORFs) based on the presence of ribosome footprints, 3-nt periodicity on Ribo-seq reads, and a minimum level of expression of the cognate transcript (Fragment per kilobase exon per million reads; FPKM ≥ 1).

In other eukaryotes, uORFs are not only prevalent, but also regulate translation of cognate downstream CDSes [20, 28]. Consistent with the reported uORF-mediated repression of translation at canonical CDSes [46–48], we observed individual examples of highly-translated uORFs upstream of their cognate lowly-translated CDSes (Fig. 4b-c). Because, sequence and mRNA secondary structure can modulate translation [49–51], we performed linear regression with these features against translation efficiency in *Toxoplasma*, as previously described [28]. Briefly, we used annotated *Toxoplasma* CDSes lacking uORFs as a training set to define the sequence motif that promotes translation initiation (initiation context), by weighting the contribution of position-specific scoring matrix (PSSM) to translation efficiency of individual transcripts (Fig. 4d). Next, we used the PSSM to score initiation sequences in individual transcripts that contain uORFs (weighted relative entropy, WRENT) (See Methods). Relative to canonical CDSes, WRENT scores at uORFs were generally unfavourable to translation initiation (Fig. 4e). We then calculated the secondary structure ensemble free energy [52], using the ViennaRNA package [53], in a 35-nt sliding window across entire transcripts to evaluate the effect of mRNA secondary structure on translation. Unlike humans and mice [28], *Toxoplasma* transcripts exhibited an unstable secondary structure before the CDS start codon and a more stable secondary structure after the CDS start codon (Fig. 4f). Moreover, the stability of the secondary structure at these regions correlates with translation efficiency of the transcripts (Fig. 4g, and Additional file 3: Figure S2 and Additional file 4: Table S1). Thus, most uORFs in *Toxoplasma* are not efficiently translated and mRNA secondary structure putatively regulate translation efficiency in *Toxoplasma*.

## Discussion

During the lytic cycle, *Toxoplasma* frequently transitions between an intracellular and extracellular niche, that is characterized by a variety of molecular changes in the parasite, including distinct transcriptional profiles [54]. Although components of the translation initiation complex, such as eIF2α, reportedly modulate stress response, extracellular survival and, virulence in *Toxoplasma* [23, 26], global translational changes during *Toxoplasma* lytic cycle are largely unknown. Here, we used ribosome profiling to reveal that translational regulation of gene expression is prominent during *Toxoplasma*s’ lytic cycle*.* Additionally, our data suggest mRNA secondary structure potentially regulate translation in *Toxoplasma*. Even though most of the genes expressed during the lytic cycle are known to exhibit a cyclic expression pattern coinciding with the different cell cycle stages [55], we show that the expression and translation of most of these genes are not temporally or spatially synchronized during the lytic cycle. However, since parasite replication and egress is not synchronized among individual parasites, it is impossible to decipher the translational changes that occur as the parasite adapts to the extracellular microenvironment. Thus, it is not clear whether the differences in translation efficiencies between intracellular and extracellular parasites observed in this study are maintained throughout the lytic cycle. With single-cell or time course analysis of the *Toxoplasma* “translatome”, we may be able to show fluctuations in translation as the parasites egress or re-infect host cells.

Interestingly, *Toxoplasma* transcripts exhibited less stable RNA secondary structure before the ATG start site. Similar reduction in RNA secondary structure have been reported in zebrafish [28]. In contrast to CDSes, this switch from unstable to stable secondary structure around the initiation codon was not observed in uORFs. This distinction in the initiation context of uORFs and CDSes, in terms of both sequence and secondary structure suggests that these two features are important for start site selection in *Toxoplasma*. uORFs have been shown to be prevalent and regulatory in a variety of organisms, including Apicomplexans [21, 56]. However, their prevalence and regulatory potential in *Toxoplasma* is largely unknown. We show that, while uORFs are prevalent in *Toxoplasma*, their translation is not favoured, probably due to selection at their initiation contexts (sequence and secondary structure). Nevertheless, we observed individual cases where high translation at an uORF correlates with weak translation at a cognate downstream CDS, which raises interesting questions that are worthy of further investigations. For example, is the translation of uORF unfavourable at all the developmental stages? How is the translation of uORFs regulated in *Toxoplasma*? Additionally, the mechanisms that regulate translation efficiency in *Toxoplasma*, which are equivocal, are worthy of further investigation. High ribosome occupancy may not be related to high rates of translation but rather ribosome pausing [57], which can be caused by long stretches of rare codons, high mRNA secondary structure, or interactions of the growing polypeptide chain with the ribosome [58, 59]. Overall, it is worthy investigating the role of translational control in modulating *Toxoplasma* strain differences in virulence, adaption to variable host genetic background or host cell activation status.

## Conclusion

The results presented in this work reveals key aspects of translational control in *Toxoplasma gondii* during the lytic cycle. We show that many dysregulated genes are translationally regulated during intercellular parasite transmission and that uORFs are prevalent, although not translationally favoured in *Toxoplasma gondii*. We anticipate that this work will be the basis for future research on translational regulation in the different development stages of the parasite and host cell microenvironments.

## Methods

### Parasite culture, ribosome isolation and, sequencing libraries

*Toxoplasma gondii* was maintained in the laboratory by serial passage on human foreskin fibroblasts (HFFs), according to standard procedures [36]. For ribosome profiling, HFF monolayers in T175 flasks were infected with a high inoculum of a type I (RH) *Toxoplasma* strain. After 2 h of infection, the cell culture medium was removed, the monolayer rinsed with ice cold Phosphate saline buffer (PBS) to remove any extracellular parasites, fresh cell culture medium added, and the parasites let to replicate and lyse for ~18 h. 10 mins before harvest, cyclohexamide (100 μg/ml) was added to the cell culture. Cell culture supernatant, containing lysed out extracellular parasites, was harvested and passed through 5 μm filters to remove HFFs. The remaining HFF monolayer, containing intracellular parasites, was rinsed with PBS to remove any extracellular parasites, scrapped, syringe lysed using 27G needles, and passed through 5 μm filters. The parasites were pelleted by centrifugation at 1700 × g, 4 °C for 7 min. The parasite pellets (intracellular and extracellular) were washed with polysome buffer and processed for ribosome profiling, as previously described [4].

### Pre-processing of Ribo-seq and RNA-seq data

Ribo-seq and RNA-seq reads were stripped from adapter sequences and aligned to the *GT1 Toxoplasma* genome (v28) using the split-aware aligner STAR [60], allowing up to 4 mismatches and discarding reads shorter than 20 nt. P-site locations and read length off-sets were inferred from the Ribo-seq data as previously described [3]. Normalized read counts (NRC) values for Ribo-seq and RNA-seq data were calculated in DESeq2 [61] based on counts data generated using HTseq [37]. P-site positions, RNA-seq coverage, RNA-site positions for different de novo assembled gene structures were created in RiboTaper [3]. All the raw and processed data can be obtained from NCBI using the are GEOarchive accession number GSE99395.

### Exon level annotation and ORF identification

First, GT1 transcripts were reconstructed *de novo* in Trinity [34] and PASA [62] guided by the GT1 genome (ToxoDB v28) [32]. Next, we used RiboTaper to identify ORFs as previously described [3]. Briefly, we used the annotated canonical coding sequences (CCDS) in ToxoDB to distinguish de novo assembled exons that; 1) overlap annotated exons in ToxoDB (CCDS), 2) do not overlap any exons inside CCDS-containing genes (non-CCDS) and, 3) overlap non-CCDS containing genes or do not overlap any annotated gene (non-CCDS). The non-CCDS included novel 5′/3′ UTRs, alternatively spliced exons, and novel exons. Next ORFs were defined based on the presence of an AUG start codon and an in-frame stop codon, after training the pipeline with 1000 CCDS from ToxoDB and random shuffling of the P-sites. Next, every transcript with a pair of consecutive start-stop codons (ORFs) was tested for 3-nt periodicity (*P* ≤ 0.05) and all ORFs with less than 50% of in-frame P-sites discarded (Refer to [3] for a detailed description of the RiboTaper pipeline). Translation initiation context, RNA-secondary structure, upstream open reading frames (uORFs) repressiveness and, uORF positional frequencies and biases were identified and modelled as previously described [28].

## Additional files

Additional file 1: Figure S1. 3-nucleotide periodicity of ribo-seq reads as predicted in the Bioconductor package, Riboprofiling for all reads and 26–30-nt reads. The numbers in red show read offsets from the AUG start site. (AI 145 kb)

Additional file 2: Dataset. A) Average DESeq2 normalized RNA-seq and Ribo-seq read counts for all the transcripts tested for differential expression and ribosome occupancy. B) Genes exhibiting low ribosome occupancy but above background mRNA abundance. C) Genes exhibiting low mRNA abundance but above background ribosome occupancy. D) Genes that are differentially regulated both at the level of mRNA abundance and ribosome occupancy (RNA + RIBO). E) Genes that are differentially regulated only at the level of mRNA abundance (RNA-Only). F) Genes that are differentially regulated only at the level of ribosome occupancy (RIBO-Only). G) Genes that exhibit significant differences in translation efficiency between in intercellular and extracellular parasites. (XLS 4356 kb)

Additional file 3: Figure S2. A scatter-plot of the correlations between secondary EFE and CDS TE in all filtered transcripts. (AI 7943 kb)

Additional file 4: Table S1. Individual correlations of sequence features with CDS TE for various transcript sets. (DOC 36 kb)

## Abbreviations

CCDS: Canonical coding DNA sequences
dORF: Downstream Open Reading Frame
FDR: False Discovery Rate
HFF: Human foreskin fibroblasts
NRC: Normalized Read Counts
ORF: Open reading frame
PASA: Program to Assemble Spliced Alignments
PBS: Phosphate Buffered Saline
PSSM: Position-specific scoring matrix
SME: Standardized major-axis estimation
TE: Translation efficiency
uORF: Upstream open reading frame
UTR: Untranslated region
WRENT: Weighted relative entropy
