# Supplementary material for: Comparative ribosome profiling uncovers a dominant role for translational control in Toxoplasma gondii
Source: BMC Genomics. 2017 Dec 11;18:961. doi: 10.1186/s12864-017-4362-6 (PMC5725899; doi:10.1186/s12864-017-4362-6)
Supplement: Supplementary file 4 — Individual correlations of sequence features with CDS TE for various transcript sets. (DOC 36 kb) [file 12864_2017_4362_MOESM4_ESM.doc]

**Table S1** Individual correlations of sequence features with CDS TE for various transcript sets

|  | **All filtered transcripts** | | | **Filtered transcripts with uORFs** | | | **Filtered transcripts without uORFs** | | |
| --- | --- | --- | --- | --- | --- | --- | --- | --- | --- |
|  | Pearson r | *p value* | Fold change | Pearson r | *p value* | Fold change | Pearson r | *p value* | Fold change |
| **Gene Expression (log10 FPKM)** | 0.1643 | 1.09E-23 | 1.688 | 0.1685 | 4.69E-21 | 1.675 | 0.1546 | 1.42E-05 | 1.544 |
| **log10 uORF density** | -0.1379 | 4.22E-17 | 0.6642 | -0.1960 | 4.56E-28 | 0.5567 | NA | NA | NA |
| **5' leader mean sec struct EFE** | 0.1331 | 6.20E-16 | 1.541 | 0.1371 | 2.21E-14 | 1.551 | 0.1449 | 4.50E-04 | 1.525 |
| **5' leader log10 length** | 0.1054 | 1.44E-10 | 1.371 | 0.1078 | 1.97E-09 | 1.399 | 0.2379 | 3.55E-09 | 1.908 |
| **CDS mean sec struct EFE** | 0.0684 | 3.27E-05 | 1.236 | 0.0593 | 9.9E-04 | 1.202 | 0.1245 | 2.23E-03 | 1.447 |
| **CDS start sec struct EFE right** | -0.06398 | 1.02E-04 | 0.8248 | -0.0548 | 2.4E-03 | 0.8456 | -0.1071 | 8.61E-03 | 0.7369 |
| **CDS start sec struct EFE left** | 0.1405 | 1.41E-17 | 1.507 | 0.1373 | 2.02E-14 | 1.497 | 0.1558 | 1.66E-04 | 1.532 |
| **CDS WRENT score** | 0.1189 | 4.62E-13 | 1.443 | 0.1134 | 2.76E-10 | 1.422 | 0.1471 | 2.97E-04 | 1.523 |
